# Deciphering the role of *DOT1L* germline pathogenic variants in familial serrated polyposis syndrome

**DOI:** 10.1016/j.gendis.2026.102235

**Published:** 2026-05-12

**Authors:** Gemma Llargués-Sistac, Yasaman Sadat Keshmiri, Irene Boluda-Luis, Jenifer Muñoz, Teresa Ocaña, Stefan Aretz, Daniel Rodríguez-Alcalde, DOT1L germline predisposition to serrated polyposis syndrome consortium, Leticia Moreira, Sergi Castellví-Bel

**Affiliations:** a Gastroenterology, August Pi i Sunyer Biomedical Research Institute (IDIBAPS), CIBEREHD, Hospital Clínic Barcelona, Barcelona 08036, Spain; b Faculty of Medicine and Health Sciences, University of Barcelona (UB), Barcelona 08036, Spain; c Institute of Human Genetics, Medical Faculty, University of Bonn, Bonn 53127, Germany; d National Center for Hereditary Tumor Syndromes, University Hospital Bonn, Bonn 53127, Germany; e Digestive Diseases Section, Móstoles University Hospital, Móstoles, Madrid 28935, Spain

Serrated polyposis syndrome (SPS) is characterized by numerous and/or large serrated polyps and increased colorectal cancer risk.^1^ The underlying etiology for SPS is largely unknown. In a previous study, we identified disruptor of telomeric silencing 1-like (*DOT1L*) as a candidate gene for SPS germline predisposition.^2^ This finding was subsequently validated in an independent cohort of 211 SPS patients. Two rare and potentially pathogenic variants in *DOT1L* were detected. *DOT1L* encodes the only lysine methyltransferase that catalyzes mono-, di-, and tri-methylation of histone H3 at lysine 79 (H3K79). Given that aberrant methylation is a hallmark of the colorectal cancer serrated pathway, the aim of this study was to functionally characterize the identified variants.

Despite being the most frequently identified polyposis syndrome, its underlying causes remain among the least understood. Clinical diagnosis relies on established criteria outlined by the WHO, first established in 2010 and updated in 2019, including: i) ≥ 5 serrated lesions/polyps proximal to the rectum, with all being ≥ 5 mm in size and at least 2 being ≥ 10 mm in size; ii) > 20 serrated lesions/polyps of any size distributed throughout the large bowel, with ≥ 5 being proximal to the rectum.^1^

SPS is associated with a significantly increased risk of colorectal cancer, yet its hereditary etiology remains elusive, and only ring finger protein 43 (*RNF43*) has been proposed as a candidate gene, although with controversy.^3^ A previous germline whole-exome sequencing study from our research group focusing on cases with familial SPS aggregation proposed 14 novel candidate genes for germline SPS predisposition.^2^

The serrated pathway to colorectal cancer is primarily characterized by the presence of *BRAF* mutations and global promoter hypermethylation across the genome. *DOT1L* encodes a non-SET domain methyltransferase involved in the regulation of gene expression through methylation of H3K79. Initially identified in *Saccharomyces cerevisiae*, *DOT1L* is evolutionarily conserved, underscoring its functional relevance. Notably, it is the only methyltransferase capable of catalyzing mono-, di-, and tri-methylation of H3K79 in a non-processive manner within the histone globular domain.^4^

The discovery cohort consisted of 16 families, with a total of 39 SPS patients.^2^ For replication, 211 additional SPS cases were collected from high-risk colorectal cancer clinics. An additional external SPS cohort (*n* = 184) with available sequencing data for unrelated SPS patients was also analyzed. Detailed methodologies, including next-generation sequencing, loss of heterozygosity, and mutational signature analyses, are provided in the supplementary data.

Functional characterization of *DOT1L* variants was performed in two different cell lines (human colorectal SW837 cells and human HAP1 cells). Specific variants were introduced using CRISPR-Cas9 knock-in strategies. A synonymous variant (not changing amino acid, nor affecting splicing) was included as an internal control. Cells were synchronized at prometaphase using nocodazole. Experimental procedures, including RNA extraction, quantitative PCR, flow cytometry, protein extraction, and Western blotting, are described in the supplementary data.

In the discovery cohort, we identified a heterozygous missense variant in *DOT1L* (NM_032482.3), c.3307G>A (p.Val1103Met). This variant was found in two siblings diagnosed with SPS at ages 49 and 43, as well as in their mother (MOS_SPS_11; supplementary data; Fig. S1), also diagnosed with colorectal cancer at age 54. In the validation cohort, a second heterozygous potentially pathogenic missense variant in *DOT1L*, c.3475G>C (p.Asp1159His), was identified in an unrelated SPS patient (PanSPS_065). The individual was diagnosed with SPS at age 58 and with lung cancer at age 60 (supplementary data; Fig. S1). Her family history was notable for skin cancer in her mother at age 63, as well as prostate cancer in other relatives. The location of the identified variants within the DOT1L protein is shown in Figure 1A. Further details for these two *DOT1L* variants and the patient’s tumor phenotype can be found in supplementary data (Fig. S2 and S6; Table S3). Single-cell clones derived from nucleofected pools in which the variant of interest had been successfully introduced were selected, and *DOT1L* expression at both the mRNA and protein levels was assessed (supplementary data; Fig. S3).

**Figure 1:**
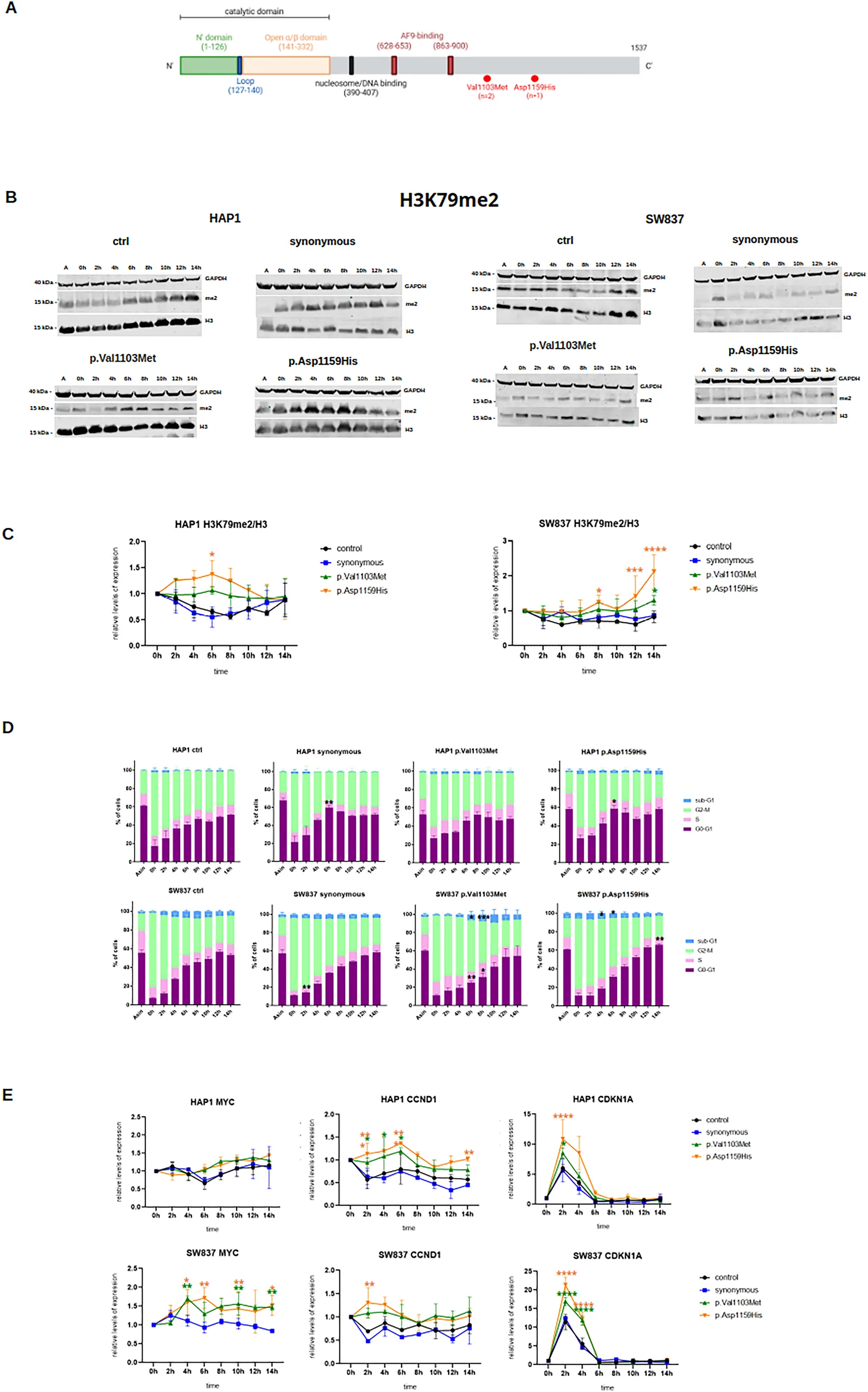
Functional characterization of *DOT1L* germline variants and their impact on H3K79 methylation and cell cycle progression. **(A)** Schematic representation of the DOT1L protein structure. The panel shows the structure of DOT1L with the location of the identified germline variants (p.Val1103Met and p.Asp1159His) relative to the main functional domains. The numbers indicate the total number of independent SPS cases identified for each variant across all cohorts. **(B)** Cell cycle-dependent dynamics of H3K79 di-methylation by DOT1L. Western blotting analysis of H3K79me2 levels in HAP1 and SW837 cells (wild-type control, synonymous variant, and *DOT1L* variants). Cells were synchronized at prometaphase with nocodazole and collected at the indicated time points post-release (0–14 h). Asynchronous (As) cells are shown for baseline comparison. Blots were probed with antibodies against H3K79me2 and total H3. GAPDH and total H3 served as loading controls. **(C)** Quantitative analysis of H3K79me2 levels. The panel shows densitometric analysis of H3K79me2 normalized to total H3. Values were expressed relative to the 0 h time point for each condition. Data represent the mean ± standard error of the mean (SEM) of three independent biological replicates (*n* = 3). Statistical significance was determined using a paired *t*-test (∗*P* ≤ 0.05, ∗∗*P* ≤ 0.01, ∗∗∗*P* ≤ 0.001, and ∗∗∗∗*P* ≤ 0.0001). Wild-type cell lines (black: control), cells with a synonymous (blue), p.Val1103Met (green), and p.Asp1159His (yellow) variants are shown. **(D)** Cell cycle progression following nocodazole synchronization. The panel shows the distribution of HAP1 and SW837 cells expressing *DOT1L* variants (synonymous, p.Val1103Met, and p.Asp1159His) and wild-type controls across cell cycle phases (G1, S, and G2/M) at multiple time points post-nocodazole release (0–14 h) by flow cytometry. Data are presented as the percentage of the total population (mean ± SEM, *n* = 3). Statistical significance between variants and controls was assessed using two-way ANOVA (∗*P* ≤ 0.05, ∗∗*P* ≤ 0.01). **(E)** Transcriptional impact on *DOT1L* target genes. The panel shows relative mRNA expression levels of *MYC*, *CCND1*, and *CDKN1A* measured by reverse transcription-quantitative PCR and normalized to *GAPDH*, in HAP1 and SW837 cells expressing *DOT1L* variants (synonymous, p.Val1103Met and p.Asp1159His) and wild-type control (ctrl). Data show the mean ± SEM of three independent experiments. Statistical significance was determined using two-way ANOVA (∗*P* ≤ 0.05, ∗∗*P* ≤ 0.01, ∗∗∗*P* ≤ 0.001, and ∗∗∗∗*P* ≤ 0.0001).

To evaluate the potential impact of *DOT1L* variants, we assessed H3K79me2 levels as a readout of *DOT1L* activity. Given that H3K79 methylation is dynamically regulated throughout the cell cycle, we analyzed its levels at multiple time points following release from nocodazole-induced arrest. Our results show that cells carrying *DOT1L* variants exhibit subtle but reproducible alterations in the temporal dynamics of H3K79me2, rather than a uniform global change. In HAP1 cells, p.Asp1159His displayed a significant increase at 6 h (*P* = 0.0150). In SW837 cells, both showed significant increases at later time points (8–14 h), particularly for p.Asp1159His at 8 h (*P* = 0.0201), 12 h (*P* = 0.0004), and 14 h (*P* = 0.0001), and for p.Val1103Met at 14 h (*P* = 0.0487) (Fig. 1C). In contrast, H3K79me3 levels remained relatively variable throughout the 0–14 h post-treatment period, showing no clear significant differences between cells with genetic variants and control cells (supplementary data; Fig. S4).

The observation that H3K79me2 differences emerge at specific cell cycle phases is consistent with the known role of *DOT1L* in cell cycle-dependent chromatin regulation, suggesting that these variants may exert their effects during defined windows of cell cycle progression.

Cell cycle progression analysis revealed a clear M-phase arrest in both HAP1 and SW837 cells. Cells carrying the variants exhibited slower progression out of arrest compared with controls (Fig. 1D).

To further investigate the impact of variants p.Val1103Met and p.Asp1159His, as well as the role of *DOT1L* in colorectal carcinogenesis, we examined the expression of *DOT1L* target genes. We evaluated the expression of several known *DOT1L*-regulated genes (*MYC*, *CCND1*, *CDKN1A*, *STAT5B*, and *CCNB1*). Among these, *MYC* and *CCND1* exhibited the most significant differences in expression between cells carrying the variants and control cells (Fig. 1E; supplementary data; Fig. S5).

While direct assessment of proliferative capacity and *in vivo* validation remain important next steps, the observed effects on cell cycle dynamics and downstream gene expression provide a mechanistic basis for how these variants may contribute to SPS pathogenesis.

In an external SPS cohort from Germany with available whole-exome sequencing data, two additional rare, protein-altering *DOT1L* variants were identified (supplementary data). This supports the overall frequency of germline *DOT1L* alterations of about 1% (4/411, 0.97%).

In cancer, *DOT1L* has been shown to play a significant role in tumorigenesis by driving the expression of genes that promote cell proliferation and survival. Germline mutations in *DOT1L* have been implicated in familial melanoma.^5^ Our results indicate that the germline *DOT1L* variants identified in SPS patients alter the temporal dynamics of H3K79 methylation during cell cycle progression, rather than inducing a global steady-state change. The location of the identified variants (p.Val1103Met and p.Asp1159His) in close proximity to the AF9-binding domains suggests that they might alter the recruitment of transcriptional cofactors. This potentially explains why the functional impact manifests as subtle temporal dynamics of H3K79 methylation rather than a complete loss of catalytic function. These observations are consistent with the observed impact on cell cycle progression and downstream target gene expression.

Together with replication in an independent external cohort, our findings support a potential role for *DOT1L* as a novel gene involved in SPS germline predisposition, with dysregulation of H3K79 methylation as a potential underlying molecular mechanism. Further studies will be required to determine the direct impact of these variants on tumor growth and *in vivo* models.

## CRediT authorship contribution statement

**Gemma Llargués-Sistac:** Writing – review & editing, Writing – original draft, Visualization, Validation, Software, Resources, Methodology, Investigation, Formal analysis, Data curation, Conceptualization. **Yasaman Sadat Keshmiri:** Writing – review & editing, Visualization, Validation, Investigation, Data curation. **Irene Boluda-Luis:** Writing – review & editing, Visualization, Validation, Investigation, Formal analysis, Data curation. **Jenifer Muñoz:** Writing – review & editing, Visualization, Validation, Investigation, Data curation. **Teresa Ocaña:** Writing – review & editing, Validation, Resources, Investigation, Data curation. **Stefan Aretz:** Writing – review & editing, Validation, Resources, Investigation, Funding acquisition, Data curation. **Daniel Rodríguez-Alcalde:** Writing – review & editing, Validation, Resources, Investigation, Data curation, DOT1L germline predisposition to serrated polyposis syndrome consortium, Writing – review & editing, Visualization, Validation, Supervision, Software, Resources, Project administration, Methodology, Investigation, Funding acquisition, Formal analysis, Data curation. **Leticia Moreira:** Writing – review & editing, Validation, Resources, Investigation, Funding acquisition, Data curation. **Sergi Castellví-Bel:** Writing – review & editing, Writing – original draft, Visualization, Validation, Supervision, Resources, Project administration, Methodology, Investigation, Funding acquisition, Formal analysis, Conceptualization.

## Ethics statement

The present study was approved by the Institutional Ethics Committee of Hospital Clínic in Barcelona (register number 2016/0867, date of approval 11/01/2017), in accordance with the Declaration of Helsinki (1975).

## Funding

GLLS was supported by a postdoctoral contract under a project funded by Asociación Española Contra el Cáncer (No. PRYGN211085CAST). XDR was supported by a contract from the Horizon Europe project STEPUPIORS (101079217; HORIZON-WIDERA-2021-ACCESS-03). JM was supported by a contract from CIBERehd. GMP was supported by a contract from IMPaCT_VUSCan (No. PMP22/00064), funded by the Instituto de Salud Carlos III (ISCIII) and by NextGenerationEU/The Recovery and Resilience Facility (RRF). YSL was supported by an INPhINIT fellowship (No. LCF/BQ/DI18/11660058) from “la Caixa” Foundation (ID 100010434) funded by the EU Horizon 2020 programme. MDG was awarded a fellowship within the “Generación D” initiative, Red.es, Ministerio para la Transformación Digital y de la Función Pública, for talent attraction (No. C005/24-ED CV1), funded by the European Union NextGenerationEU funds, through PRTR. CIBEREHD and CIBERONC (No. CB16/12/00234) are funded by the Instituto de Salud Carlos III. This research was also supported by grants from Fondo de Investigación Sanitaria/FEDER (No. 20/00113, 20/00226, INT21/00040, 23/00189), La Marató TV3 2019 (No. 202008-10), Asociación Española Contra el Cáncer (No. PRYGN211085CAST), COST Action CA17118, “la Caixa” Foundation (agreement LCF/PR/SP23/52950009), Spanish Ministry of Science and Innovation (Agencia Estatal de Investigación/FEDER; No. PID2020-112595RB-I00), CERCA Programme (Generalitat de Catalunya) and Agència de Gestió d’Ajuts Universitaris i de Recerca (Generalitat de Catalunya) (No. 2021 SGR 01185, 2021 SGR 00716, 2021 SGR 01112). This work is supported (not financially) by the European Reference Network on Genetic Tumor Risk Syndromes (ERN GENTURIS). ERN GENTURIS is funded by the European Union.

## Conflict of interests

MDG declares a European patent application with application number EP25305077.7. Other authors have nothing to disclose.
