# Supplementary material for: Deciphering the role of DOT1L germline pathogenic variants in familial serrated polyposis syndrome
Source: Genes Dis. 2026 May 12;14(2):102235. doi: 10.1016/j.gendis.2026.102235 (PMC13634565; doi:10.1016/j.gendis.2026.102235)
Supplement: Supplementary file 1 [file mmc1.docx]

**SUPPLEMENTARY DATA**

***DOT1L* germline predisposition to serrated polyposis syndrome consortium**

Gemma Llargués-Sistac, Yasaman Sadat Keshmiri, Irene Boluda-Luis, Laia Bonjoch, Jenifer Muñoz, Aina Aguayo, Gerard Muñoz-Pujol, Yasmin Soares de Lima, Xavier Domínguez-Rovira, Marcos Díaz-Gay, Teresa Ocaña, Miriam Cuatrecasas, Sandra López-Prades, Sabela Carballal, Guerau Fernàndez, Isabel Spier, Anna K. Sommer, Stefan Aretz, Luis Bujanda, Gabriel Capellá, Laura Valle, Joaquín Cubiella, Daniel Rodríguez-Alcalde, Silvia Carles-González, Fatima Pérez-Rial, Clara Ruiz-Ponte, Antoni Castells, Francesc Balaguer, Leticia Moreira, Sergi Castellví-Bel.

**Supplementary Materials and Methods**

The discovery cohort consisted of 16 families, with a total of 39 SPS patients.^1^ For replication, 211 additional SPS cases were collected from high-risk CRC clinics at Hospital Clínic de Barcelona, Institut Català d’Oncologia-IDIBELL, and Fundación Pública Galega de Medicina Xenómica. Germline DNA was extracted from peripheral blood mononuclear cells using the QIAamp DNA Blood kit (Qiagen, Hilden, Germany), as per manufacturer’s instructions. An additional external SPS cohort (n = 184) with available sequencing data for unrelated SPS patients from the University of Bonn and the Medical Genetics Center Munich in Germany was also accessed. Patients did not present with pathogenic variants in *MUTYH*, *APC*, *RNF43* or the DNA MMR genes, when analyzed using gene-panel sequencing and screening for point mutation, copy-number variants and potential splicing alterations. Since the recruitment occurred prior to the release of the current SPS guidelines, both the discovery and replication cohorts were selected based on the 2010 WHO criteria.^2^ Written informed consent was obtained in all cases. The present study was approved by the Institutional Ethics Committee of Hospital Clínic in Barcelona (register number 2016/0867, date of approval 11/01/2017), in accordance with the Declaration of Helsinki (1975).

Whole-exome sequencing for the discovery cohort was performed using the HiSeq2000 Platform (Illumina, San Diego, USA). Exon capture and enrichment were accomplished with the SureSelectXT All Exon v5 kit (Agilent, Santa Clara, CA, USA). Sequencing library quality control was assessed using the Bioanalyzer 2000 (Agilent, Santa Clara, CA, USA). The samples were indexed, pooled, and sequenced in parallel with a paired-end 2×100 base pair read length protocol. For additional information on the sequencing process and quality control, refer to Soares de Lima et al.^1^ For replication purposes, a panel of 20 genes was designed for the replication cohort using the DesignStudio online tool (Illumina, San Diego, USA), incorporating the 14 candidate genes identified in the discovery cohort analysis (*ANXA10*, *ASXL1*, *CFTR*, *DOT1L*, *HIC1*, *INO80*, *KLF3*, *MCM3AP*, *MCM8*, *PDLIM2*, *POLD1*, *TP53BP1*, *WNK2*, and *WRN*), as previously reported.^3^ Additional genes were included based on existing literature or previous recommendations from our research group (*ANXA1*, *ANXA2*, *ASLX2*, *POLE*, *FBLN2*, and *RNF43*). To validate specific genetic variants, Sanger sequencing (Eurofins Genomics) was employed. Discovery and replication cohorts underwent the same variant prioritization strategy.^1,3,4^ Emphasis was placed on rare (allele frequency ≤ 0.1% according to gnomAD, <https://gnomad.broadinstitute.org/>), nonsynonymous, truncating, or missense variants found in the canonical transcripts that met at least three of six pathogenicity prediction tools (PhyloP, SIFT, PolyPhen, MutationTaster, CADD, and LRT). Additionally, we prioritized genes with germline variants identified in both cohorts that may be functionally relevant to SPS, CRC, or other cancer types.

The ALFRED (allelic loss featuring rare damaging) method was applied to test Knudson’s two-hit hypothesis across the genome, as described in previous work.^1^ Somatic tumor mutational burden (TMB) and mutational signatures were identified using the SigProfilerAssignment tool (COSMIC v3.4 reference mutational signatures for hg19/GRCh37).^5,6^ To assess loss of heterozygosity (LOH), Sanger sequencing (Eurofins Genomics) was used to compare wild-type and tumor DNA samples to detect a reduction in the reference allele.

Functional characterization of *DOT1L* genetic variants

*Cell lines*

Human colorectal SW837 cells (cat. no. 91031104, ECACC; Sigma Aldrich) were cultured in RPMI-1640 medium (Gibco, Waltham, MA) supplemented with 10% fetal bovine serum (Gibco, Waltham, MA). Human HAP1 was kindly provided by G. Muñoz-Pujol. HAP1 cells were grown in Iscove's Modified Dulbecco's Medium (IMDM; CYTIVA, Marlborough, MA, USA), containing L-Glutamine and 25 mM HEPES and supplemented with 10% fetal bovine serum (Gibco, Waltham, MA). Both cell lines were cultured at standard growth conditions of 37°C and 5% carbon dioxide (CO2) and routinely tested for mycoplasma contamination using the Mycoplasma Gel Detection kit (Biotools, Madrid, Spain).

*Generation of knock-in cell line models using CRISPR-Cas9*

The main objective of this study was to set up a protocol to generate new colorectal cancer cell line models in which the pathogenicity study of genetic variants could be assessed without the need to introduce plasmids in the genome of the cells.

The introduction of specific genetic variants using CRISPR-Cas9 technology requires the design of a CRISPR RNA (crRNA) for each variant, which targets a specific genomic region. Co-delivery of a single-stranded oligonucleotide (ssODN) repair template carrying the desired mutation enables precise editing through the homology-directed repair (HDR) pathway. Each Cas9-ribonucleoprotein (RNP) complex (crRNA:tracrRNA-Cas9) was delivered into cells via nucleofection.

KI cell line models with the genetic variants of interest were generated following the protocol previously reported by Muñoz-Pujol et al.^7^ All CRISPR-Cas9 reagents were designed and obtained from Integrated DNA Technologies (IDT, Coralville, IA, USA). The delivery process and off-target analysis were performed in accordance with IDT's guidelines (<https://eu.idtdna.com>). Details of the crRNAs, ssODNs and primers used in this study can be found in **Supplementary Table 1**.

The RNP complex was formed by heating the oligos at 95°C for 5 minutes, cooled at room temperature and combined with an Alt-R S.p. Cas9 Nuclease V3. The mixture was incubated at room temperature for up to 20 minutes. Nucleofection was performed using the Neon Transfection System (MPK5000, Thermo Fisher Scientific, Waltham, MA, USA). For each electroporation, 4×10⁵ HAP1 cells and 8×10⁵ SW837 cells were resuspended in Resuspension Buffer R (MPK1096, Thermo Fisher Scientific, Waltham, MA, USA). To this, the RNP complex and the ssODN were added. The mixture of cells and RNP was nucleofected in a total volume of 10 μL at 1750 V, with a 10 ms pulse width and 3 pulses for HAP1, and 1650V, with a 10 ms pulse width and 3 pulses for SW837. After nucleofection, cells were seeded into 6-well plates with pre-warmed complete IMDM and HDR enhancer V2. The nucleofected cells were allowed to recover and expand. Sanger sequencing (Eurofins Genomics) was performed on pooled cells to verify CRISPR-Cas9 editing. Limiting dilution was applied to select single-cell clones. Clones displaying DOT1L expression levels comparable to wild-type controls were prioritized for subsequent analyses. In the HAP1 cell line, clone 3 was chosen for the p.Val1103Met variant and clone 26 for p.Asp1159His. In the SW837 cell line, clones 19 and 15 were selected for the p.Val1103Met and p.Asp1159His variants, respectively.

In addition to the two *DOT1L* genetic variants chosen for the study, we decided to include a synonymous variant (not changing amino acid, not affecting splicing) to use as an internal control for the CRISPR-Cas9 technique. The same guide to generate the variants was used with a HDR template with a synonymous amino acid change: c.3309G>A (p.Val1103Val) and c.3477G>T (p.Asp1159Asp).

*Cell synchronization*

SW837 and HAP1 cells were synchronized at prometaphase by treatment with nocodazole (0.2 µg/ml and 0.02 µg/ml, respectively), for 16 hours. Nocodazole (31430-18-9, Merck) is a microtubule depolymerizing agent that disrupts microtubule dynamics, arresting cells in prometaphase of the cell cycle. After the 16h incubation, the agent was removed by washing with PBS, and cells placed with fresh medium to promote progression into the next phase of the cell cycle. Cells were collected every two hours post-treatment release, from 0h to 14h.

*RNA extraction and Quantitative Real-time PCR*

Total RNA was extracted using the RNeasy Mini Kit following manufacturer’s instructions (Qiagen, Hilden, Germany) and quantified with a Nanodrop spectrophotometer (ThermoFisher, Waltham, MA). Reverse transcription was carried out using the High-Capacity cDNA Reverse Transcription Kit (Applied Biosystems, Foster City, CA). Gene expression of genes of interest was performed on the QuantStudio 1 System (Applied Biosystems, Foster City, CA), using the TaqMan Universal Master Mix II (Applied Biosystems, Foster City, CA) and TaqMan probes for *MYC*, *CDKN1A*, *CCND1*, and *GAPDH* (details provided in **Supplementary Table 2**). GAPDH served as the reference gene for normalization, and relative quantification was calculated using the -∆∆Ct method.

*Flow cytometry*

Flow cytometry analysis was used to assess the distribution of cells across different cell cycle phases. SW837 and HAP1 cells collected at 0-14h post-nocodazole treatment were washed twice with ice-cold PBS and fixed with ice-cold 70% ethanol and incubated with RNase inhibitor (Applied Biosystems) at 37°C for 30 minutes. Afterward, propidium iodide (PI) was added to a final concentration of 50 μg/ml for DNA staining. A total of 20,000 fixed cells were analyzed using a 3-laser FACSCanto II (BD Biosciences, Eysins, Switzerland). The cell cycle distribution was then processed and analyzed using FlowJo software. The percentage of cells in each cell cycle phase was determined relative to the total number of cells.

*Protein extraction and Western Blot*

Cells were lysed with RIPA buffer (Sigma-Aldrich, Burlington, MA), which was supplemented with cOmplete Protease Inhibitor Cocktail and PhosSTOP (Roche, Basel, Switzerland). Pierce BCA Protein Assay Kit (ThermoFisher Scientific) was used for total protein quantification, according to manufacturer’s instructions. For both cell lines, 100µg of total protein extracts were loaded and separated on NuPAGE™ gels according to the manufacturer's guidelines (ThermoFisher). Large proteins (DOT1L, vinculin) were separated on NuPAGE™ 3-8% gels using Tris-Acetate SDS Running Buffer (20X); whereas NuPAGE™ 12% gels and MES SDS Running Buffer (20X) were used for smaller proteins (H3, H3K79).

Proteins were subsequently transferred to PVDF membranes (Millipore, Bedford, MA). The blots were incubated with primary antibodies against H3K79me2, H3K79me3, Histone H3, DOT1L, GAPDH and vinculin. Either 800CW anti-mouse or 680RD anti-rabbit IRDye secondary antibodies were used based on the host species of the primary antibody. Details of the antibodies used can be found in **Supplementary Table 2**. Protein detection was performed using the Odyssey Imaging System (LI-COR, Lincoln, NE), and the data analyzed with Image Studio 4.0 software.

*Protein structure predictions*

The three-dimensional (3D) structure of the DOT1L protein was predicted using AlphaFold (<https://alphafoldserver.com/>). AlphaMissense is an AI model that builds on AlphaFold used to estimate the likelihood of a variant being pathogenic.

**Table S1.** crRNAs, ssODNs and primers used in this study

| **Gene** | **Genetic variant** | **crRNA (5'-3')** | **ssODN (5'-3')** | **Forward primer (5'-3')** | **Reverse primer (5'-3')** |
| --- | --- | --- | --- | --- | --- |
| DOT1L | c.3307G>A | GGGACGCCCAGCTTGAGCGC | CGCGGCCGGCGGAAGCGAGCATCTGCGGGGACGCCCAGCTTGAGCGCAGGCATGTCCCCCAAGCGCCGAGCCCTGCCGTCCGTCGCTGGCCTTTTCACACAGC | CAAGCAGTCGCCCTCCAGCAAG | CTGCCGGTCCCCATCCTTACCA |
|  | c.3475G>C | GAGCTCCCCTGTGCCCTACC | GTCCCCGGAGACCTCCCTGAAGAGCTCCCCTGTGCCCTACCAGCACCACGACCAGCCCCCCGTGCTCAAGAAGGAGCGGCCTCT | GCTGGGCAGGGCATCAGTTTCC | ACACAGTGAGCAGGCACCTCCT |

**Table S2.** List of antibodies and Taqman probes used in this study.

| **ANTIBODIES** | | |
| --- | --- | --- |
| **Target** | **Manufacturer** | **identifier** |
| Histone H3 | Cell signaling | 4499 |
| Histone H3 (di methyl K79) | Abcam | ab3594 |
| Histone H3 (tri methyl K79) | Abcam | ab2621 |
| KMT4 / DOT1L | Abcam | ab64077 |
| GAPDH | Cell signaling | 2118 |
| Vinculin | Santa Cruz | sc-73614 |
| IRDye 680RD Goat anti-Rabbit IgG Secondary Antibody | LI-COR Biosciences | 925-68071 |
| IRDye 800CW Goat anti-Rabbit IgG Secondary Antibody | LI-COR Biosciences | 926-32211 |
| IRDye 800CW Goat anti-Mouse IgG Secondary Antibody | LI-COR Biosciences | 925-32210 |

| **TaqMan probes** | | |
| --- | --- | --- |
| **Target** | **source** | **identifier** |
| MYC | Thermofisher | Hs00153408_m1 |
| CDKN1A | Thermofisher | Hs00355782_m1 |
| CCND1 | Thermofisher | Hs00765553_m1 |
| DOT1L | Thermofisher | Hs01579928_m1 |
| STAT5B | Thermofisher | Hs00560026_m1 |
| CCNB1 | Thermofisher | Hs01030099_m1 |
| GAPDH | Thermofisher | Hs99999905_m1 |

**Supplementary Results**

**A)	MOS_SPS_11**

c.3307G>A (p.Val1103Met)

**B)	PanSPS_065**

c.3475G>C (p.Asp1159His)

**Figure S1. Pedigrees of the two SPS families harboring germline *DOT1L* variants**. **A)** Pedigree of the SPS family from the discovery cohort. **B)** Pedigree of the family from the validation cohort. Black filled symbol indicates affected with CRC (upper right quarter), SPS (lower right quarter) or other types of cancer (lower left quarter). Colon, uterine, skin and ovarian teratoma refer to the types of cancers. Ages at diagnosis are depicted. The probands are indicated by an arrow.

**A)**

**AA3559**

**DOT1L	DOT1L**

c.3307G>A	**AA3560**	c.3477G>T

p.Val1103Met	p.Asp1159His

**AA3563**

**B)	C)**

*germline*

*tumoral*

**Figure S2.** **Characterization of *DOT1L* germline variants. A)** Sanger sequencing validation of germline *DOT1L* variants identified through whole-exome sequencing in MOS_SPS_11 (p.Val1103Met) and PanSPS_065 (p.Asp1159His). **B)** Single base substitution (SBS) mutational signatures identified in each sample are shown. SBS1 and SBS5 are considered clock-like signatures associated with the accumulation of mutations over time. SBS15 and SBS21 are linked to deficiencies in DNA mismatch repair (MMR), while SBS54 is recognized as a sequencing artifact (<https://cancer.sanger.ac.uk/cosmic/signatures/SBS/index.tt>). **C)** Loss of heterozygosity (LOH) analysis in *DOT1L* variant proband from PanSPS_065. Visualization of LOH using Sanger sequencing.

**A)**

*DOT1L* WT

*DOT1L* KI

**p.Val1103Met	p.Asp1159His**

**B)**

**Figure S3. *DOT1L* knock-in cell lines validation.** A) Sanger sequencing confirming the generation of CRISPR/Cas9 knock-in cell models. Position of each variant of interest is blue marked for both non-edited cells (WT) and knock-in models (KI). B) Validation of *DOT1L* expression of HAP1 and SW837 clones generated. *DOT1L* expression was checked at the mRNA (qPCR) and protein expression level (western blot). S, synonymous variant (p.Val1103Val); WT, wild-type; V1, p.Val1103Met; V2, p.Asp1159His.

**A)	H3K79me3**

**ctrl	synonymous**

A	0h	2h	4h	6h	8h	10h 12h	14h	A	0h	2h	4h	6h	8h	10h	12h	14	GAPDH

40kDa -	GAPDH

3me

15kDa -	3me

H3

15kDa -	H3

**HAP1**

**p.Val1103Met	p.Asp1159His**

A	0h	2h	4h	6h	8h	10h	12h	14h	A	0h	2h	4h	6h	8h	10h	12h	14h

40kDa -	GAPDH	GAPDH

15kDa -	3me	3me

15kDa -	H3	H3

**ctrl	synonymous**

A	0h	2h	4h	6h	8h	10h	12h	14h	A	0h	2h	4h	6h	8h	10h	12h	14h

GAPDH	GAPDH

40kDa -	3me	1

15kDa -	3me	H3

.

15kDa -	H3

**SW837**

**p.Val1103Met	p.Asp1159His**

A	0h	2h	4h	6h	8h	10h	12h	14h	A	0h	2h	4h	6h	8h	10h	12h	14h

40kDa -	GAPDH	GAPDH

.

15kDa -	3me	3me

.

15kDa -	H3	H3

**B)**

**Figure S4. Analysis of global pattern of tri-methylation of H3K79 by DOT1L. A)** Western blot analysis of H3K79me3 of protein extracts from HAP1 and SW837 cells expressing *DOT1L* variants, wild-type *DOT1L* (control), and a *DOT1L* synonymous variant. The blots show the change in H3K79me3 protein expression at different time-points post-nocodazole treatment (0h-14h). Protein expression of asynchronous cells is depicted (A). Blots were probed with antibodies against total H3 and H3K79me3. H3 and GAPDH were used as internal loading controls. **B)** Quantitation analysis of H3K79me3 protein bands density from western blot (*p≤0.05, paired t test). H3K79me3 protein expression was corrected to H3 protein expression levels and values normalized to the expression of H3K79me3 at 0h post-nocodazole treatment for each cell line. Graphs show the comparison of global H3K79me3 protein levels from 0h to 14h post nocodazole treatment between wild-type cell lines (black: control), and cells with a synonymous (blue), p.Val1103Met (green) and p.Asp1159His (orange) variant. Values from three experiments. Significance determined by 2-way ANOVA (statistically significant; *p≤0.05)

**Figure S5. Impact of germline *DOT1L* variants on *DOT1L* downstream target genes in HAP1 and SW837 cell lines.** Relative mRNA expression levels of two *DOT1L* target genes were assessed by quantitative PCR. The graphs show the *STAT5B* and *CCNB1* mRNA expression levels of HAP1 and SW837 wild-type cells (control) and cells bearing the variants (synonymous, p.Val1103Met and p.Asp1159His), from 0h to 14h post-nocodazole treatment. Changes in mRNA expression levels are compared to 0h for each condition. Data represents the mean (±SEM) of triplicates. Expression fold change was calculated relative to *GAPDH* expression (control gene). Significance determined by 2-way ANOVA (statistically significant; *p≤0.05)

**Table S3. Rare germline genetic variants identified in the *DOT1L* gene.**

The table below summarizes the information of the variants from our discovery and validation cohort, as well as two variants found in a German international cohort. NM_006497.4 was used as reference transcript. CRC, colorectal cancer; MAF, minor allele frequency; CADD, combined annotation-dependent depletion; PolyPhen, polymorphism phenotyping.

| Variant | Family ID | Population MAF (GnomAD v.4.1.0) | Nº of homozygotes (GnomAD v.4.1.0) | CADD | PolyPhen | Study cohorts |
| --- | --- | --- | --- | --- | --- | --- |
| c.3307G>A p.Val1103Met | MOS_SPS_11 | 112/1449836 (7,725e-5) | 0 | 25.3 | damaging | Discovery |
| c.3475G>C p.Asp1159His | PanSPS_065 | 233/1461650 (1,594e-4) | 0 | 25.4 | damaging | Validation |
| c.2270G>A p.Arg757Gln | HPS_217 | 34/1453474 (1,294e-5) | 0 | 24.8 | damaging | German |
| c.3467C>G p.Pro1156Arg | MUC-GIP-0066 | 1/1461668 (6,841e-7) | 0 | 21.9 | possibly damaging | German |

*DOT1L* encodes a histone methyltransferase involved in chromatin remodeling and transcriptional regulation and has been previously implicated in colorectal tumorigenesis. Both genetic variants were validated by Sanger sequencing (**Figure 2A**). Both variants were classified as damaging by five and six out of six pathogenic prediction tools, respectively. According to the gnomAD database, the *DOT1L* gene has a LOEUF (loss-of-function observed / expected upper bound fraction) score of 0.32 and a probability of being loss-of-function intolerant (pLI) of 1, and it is predicted to be haploinsufficient (where heterozygous loss-of-function variants are not tolerated).^8^

Tumor in MOS_SPS_11 AA3563 was DNA mismatch repair proficient by immunohistochemistry. WES of tumor DNA detected a *MSH3* mutation, a TMB of 46.3 mutations per megabase, and a mutational signature profile dominated by the single-base substitution signatures SBS.1 and SBS.5, considered clock-like mutational signatures, commonly identified in CRC (**Figure 2B**).^1^ The high TMB could be due to the DNA sample being from FFPE (formalin-fixed, paraffin embedded) tissue and will not correspond to a real hypermutated case since a different mutational profile or the display of microsatellite instability will be expected. LOH was not predicted by ALFRED for either variant. Sanger sequencing analysis of advanced serrated polyp tissue from index case from PanSPS_065 corroborated the absence of LOH as predicted by ALFRED (**Figure 2C**). Sanger sequencing of the tumor sample from the proband in MOS_SPS_11 failed to amplify.

An external SPS cohort from Germany with available WES data was analyzed to identify additional rare, non-synonymous/truncating or missense *DOT1L* variants. Genetic variants were selected based on low population frequency (<0.1% in gnomAD), predicted deleterious effect on the protein, and pathogenicity according to bioinformatic prediction tools (CADD score>20). In total, two additional rare, protein-altering *DOT1L* variants were identified: c.2270G>A (p.Arg757Gln) and c.3467C>G (p.Pro1156Arg) (previous Table above).

Considering the number of variants found in all SPS analyzed cohorts, the overall frequency of germline *DOT1L* alterations in SPS could be considered ~1% (4/411, 0.97%). To contextualize these findings, we investigated the presence of rare, potentially pathogenic *DOT1L* variants in the gnomAD control population database (<0.1%; including predicted loss-of-function, missense, or in-frame indels with CADD ≥20). A total of 1,417 such *DOT1L* variants were identified in gnomAD (1,417/806,152; 0.17%). Comparatively, our SPS cohort harbored a higher frequency of rare, potentially pathogenic *DOT1L* variants, this difference being statistically significant (chi-square=14.88, *P*=0.0001).

A predicted three-dimensional structure of *DOT1L* was obtained using AlphaFold (**Fig.S6**), which also provided per-residue confidence estimates using the predicted Local Distance Difference Test (pLDDT) score, ranging from 0 to 100. The average pLDDT score for *DOT1L* was 52.44, indicating overall low model confidence. Each variant was mapped in the predicted structure (**Figure 8**). The pLDDT scores for residues harboring the variants p.Arg757Gln, p.Val1103Met, p.Pro1156Arg and p.Asp1159His were notably low (29.30, 32.60, 39.14 and 35.89, respectively), suggesting limited structural reliability at these positions. When mapped onto the predicted 3D structure, the variants were not observed to cluster spatially but appeared to be predominantly located toward the C-terminal region of the protein.

**Figure S6. Predicted 3D protein structure of DOT1L with mapped variants.** The theoretical model of the DOT1L protein shows regions of different confidence levels: light blue indicates high-confidence predictions, yellow represents low-confidence areas, and orange corresponds to regions of very low confidence. Black dots highlight the locations of the variants within the protein structure.

References

1. Soares de Lima Y, Arnau-Collell C, Díaz-Gay M, et al. Germline and Somatic Whole-Exome Sequencing Identifies New Candidate Genes Involved in Familial Predisposition to Serrated Polyposis Syndrome. *Cancers* 2021;13(4):929. <https://doi.org/10.3390/cancers13040929>
2. Snover DC, Ahnen DJ, Burt RW, Odze RD. *WHO Classification of Tumours of the Digestive System* (F. T. Bosman, F. Carneiro, R. H. Hruban, & N. D. Theise, Eds.; 4th ed., Vol. 3). IARC Publications 2010.
3. Domínguez-Rovira X, Arnau-Collell C, Gonfaus-Ortiz G, et al. Germline pathogenic variants in HIC1 DNA binding domains are associated with familial serrated polyposis syndrome. *Int J Cancer.* 2025;May 20. <https://doi.org/10.1002/ijc.35492>
4. Soares de Lima Y, Arnau-Collell C, Muñoz J, et al. Germline mutations in WNK2 could be associated with serrated polyposis syndrome. *J Med Genet.* 2023;60(6):557-567. <https://doi.org/10.1136/jmg-2022-108684>
5. Díaz-Gay M, Vangara R, Barnes M, et al. Assigning mutational signatures to individual samples and individual somatic mutations with SigProfilerAssignment. Bioinformatics 2023;39(12):btad756. <https://doi.org/10.1093/bioinformatics/btad756>
6. Sondka Z, Dhir NB, Carvalho-Silva D, et al. COSMIC: a curated database of somatic variants and clinical data for cancer. *Nucleic Acids Res.* 2024;52(D1):D1210–D1217. <https://doi.org/10.1093/nar/gkad986>
7. Muñoz-Pujol G, Ugarteburu O, Segur-Bailach E, et al. CRISPR/Cas9-based functional genomics strategy to decipher the pathogenicity of genetic variants in inherited metabolic disorders. *J Inherit Metab Dis.* 2023;46(6):1029–1042. [https://doi.org/10.1002/jimd.12681](https://doi.org/https:/doi.org/10.1002/jimd.12681)
8. Lek M, Karczewski KJ, Minikel EV, et al. Analysis of protein-coding genetic variation in 60,706 humans. *Nature* 2016;536(7616):285–291. <https://doi.org/10.1038/nature19057>
